# Empyema gall bladder and laparoscopic cholecystectomy

**DOI:** 10.4103/0972-9941.38913

**Published:** 2007

**Authors:** Iqbal Saleem Mir

**Affiliations:** Post Graduate Department of Surgery, Govt Medical College, Srinagar, Kashmir, India. E-mail: iqbalsurg@rediffmail.com

Dear sir,

I read the article about laparoscopic management of empyema gall bladder[1] with interest. Some points documented underneath need clarification as the authors have not given the details.

How was the gall bladder extracted? Was an extraction bag used?

The role of analysis by SPSS version 10 has not been further elucidated anywhere in the text. What variable was being assessed?

In the exclusion criteria did the authors exclude patients with bleeding disorders?

The USG findings discussed are non-specific and usually it is quite difficult to predict empyema gall bladder with these findings. We have found out that a thick-walled gall bladder >4 mm with a stuck stone at the neck is more of a predictor of empyema than other findings mentioned. Even the clinical features are sometimes misleading.

In table 3A the number of CBD injuries shown is 3 while as in the discussion repair of only two cases is documented. Which one is correct?

After conversion what were the results? Was cholecystectomy completed in all patients?

What is the role of intraoperative cholangiography in such cases?

What is the role of laparoscopic cholecystostomy in empyema gallbladder? Would it have decreased the overall conversion and the complication rates?

Did the very high morbidity documented justify the early intervention in pre-surgery documented cases of empyema?
